# Standards and standard-compliance

**DOI:** 10.4056/sigs.55934

**Published:** 2009-11-22

**Authors:** Peter Sterk

**Affiliations:** NERC Centre for Ecology and Hydrology, Oxford, OX1 3SR, United Kingdom

The Genomic Standards Consortium (GSC) was established in September 2005 at an international workshop in Cambridge, United Kingdom, which had and still has as its primary goal the creation of richer descriptions of our collections of genomes and metagenomes through the development of standards and tools for supporting compliance and exchange of contextual information [1]. Members of the GSC include representatives from the major sequence centers, bioinformatics centers, a range of research institutions and from the International Nucleotide Sequence Database Collaboration (INSDC). The INSDC was established some twenty years ago among DDBJ, EMBL and GenBank (http://www.insdc.org/). It took five workshops with 40-50 participants each over a period of nearly three years to produce and publish a checklist specifying the Minimal Information about a Genome Sequence (MIGS) and an extension for metagenomes (MIMS) [2]. This MIGS/MIMS checklist fits neatly on a single sheet of paper and now is a good time to reflect on whether the time, energy, effort and funds that have gone into creating this checklist has been worthwhile.

Standards are everywhere in our daily lives. The success of a particular standard depends on a number of criteria. Is it widely adopted? Are there competing standards? Can they stand the test of time? To illustrate this, here is an example of a success story: the global adoption of the Compact Cassette standard, licensed for free – albeit under pressure - by Philips in the early 1960s (http://en.wikipedia.org/wiki/Compact_cassette). It spelled the end of the then far superior reel-to-reel decks in the consumer market as the cassette players were far more affordable, in many cases portable, and tapes were exchangeable (which was not always appreciated by certain industries). Staying with magnetic tapes and the relevant equipment, there are often casualties where several standards compete. During the late 1970s and 1980s, the main video tape recording standards were VHS developed by JVC, Betamax developed by Sony and Video 2000 by Philips and Grundig. VHS came out as the winner and Betamax and Video 2000 systems disappeared from the market (http://en.wikipedia.org/wiki/Videotape_format_war). New technologies have come along, such as the Compact Disc, DVD and personal computers, which have caused a sharp decline in the sale and use of audio and video cassettes. Not surprisingly, considering how fast technologies evolve, these standards have not stood the test of time.

The aim of the GSC has been to establish a useful, widely adopted, robust, yet flexible standard for the reporting of (meta)genome contextual metadata will stand the test of time. We have made sure to learn from similar efforts by other (mainly life science) standard communities and collaborate where possible. Many of those communities are now represented under the Minimum Information for Biological and Biomedical Investigations (MIBBI) umbrella, a project that has as one of its goals “the encouragement of collaborative development between such projects, where appropriate, to avoid duplication of effort or competition” [3]. It works towards the synthesis of reporting guidelines from various communities into a suite of orthogonal standards. This is badly needed in a world where biology is changing rapidly, new technologies are emerging rapidly, and complex multi-omic experiments of the same biological samples demand good reporting standards. Unlike consumer products that come and go, much of the genomic science we do today will have value for decades to come. New sequencing technologies make new things possible and on much larger scales, too. Therefore, reporting standards should be robust, but adapt where there is demand. This can be in the form of an extension. During the GSC meeting in September 2009, the “Minimal Information about an Environmental Sequence (MIENS)” extension to MIGS/MIMS that meets the needs of communities to report any genetic marker sequence retrieved from the environment. (http://gensc.org/gc_wiki/index.php/MIENS) was finalized. The current checklist may need further adaptations for projects like the human microbiome project (http://www.hmpdacc.org/reference_genomes.php) and other projects that started after publication of the MIGS/MIMS checklist. The GSC is actively working to engage representatives of these projects to ensure coverage of areas that were not fully incorporated into MIGS/MIMS..

These facts serve as justification for creating these standards. However, how successful have our efforts been or will our efforts be? The success of a consumer product can be measured easily by looking at sales numbers and profits. The success of MIGS/MIMS will depend on how well it will be adopted by the scientific community. A small number of reports have been published where authors have included MIGS-compliant content, e.g [4], in addition to the genome reports in this journal. The Genomes Online Database (GOLD) [5] lists ongoing and finished genome projects and constitutes a  huge curation effort to include genome metadata extracted from literature. With the fast growing number of new genome projects, this will no longer be sustainable. Submissions to MG-RAST (Meta Genome Rapid Annotation using Subsystem Technology) [6] now requires the inclusion of MIGS/MIMS data. However, for the majority of genomes and metagenomes, metadata is sparse as the provision of these data are hardly ever enforced or even encouraged and supported.

This may all change soon. During the 6^th^ GSC workshop in October 2008 [7] inclusion of MIGS data in INSDC genome records was proposed. During the GSC workshop in September 2009, the representatives from GenBank at NCBI and the EMBL Bank at EBI reported on their developments in this area. It is already possible to incorporate contextual metadata in GenBank records in the form of a structured comment block that contains field names with associated values and where applicable units. Both institutes have been adapting their submission tools to support inclusion of project-specific (meta)data and will have MIGS/MIMS/MIENS-specific fields enabling submitters to supply metadata. In addition, a special MIGS keyword will be added to the genome record allowing searches through genome records filtered by MIGS-compliance. Some details are still being worked on, e.g. validation of metadata and the way the individual databases will store and represent the metadata. However, what is important is that submitters will be able to supply these data, which will be exchanged among the collaborating databases. It will hopefully encourage researchers to collect metadata at an early stage reducing the risk of losing valuable data. The adoption of the GSC MIGS/MIMS checklist by centers producing (meta)genome sequences is now a major priority for the GSC and with the INSDC behind this effort, this is an immense step forward indeed.

This development will be explored in a panel session that will be part of the GSC workshop, held during the Pacific Symposium on Biocomputing (http://psb.stanford.edu/) in January 2010 involving representatives from EMBL, GenBank and DDBJ.

## References

[1] Field D Garrity GM Morrison N Selengut J Sterk P Tatusova T Thomson N eGenomics: Cataloguing our Complete Genome Collection. Comp Funct Genomics 2005; 6: 363-368 10.1002/cfg.494 18629208 PMC2447503

[2] Field D Garrity GM Gray T Morrison N Selengut J Sterk P Tatusova T Thomson N Allen MJ Angiuoli SV The minimum information about a genome sequence (MIGS) specification. Nat Biotechnol 2008; 26: 541-547 10.1038/nbt1360 18464787 PMC2409278

[3] Taylor CF Field D Sansone SA Aerts J Apweiler R Ashburner M Ball CA Binz PA Bogue M Booth T Promoting coherent minimum reporting guidelines for biological and biomedical investigations: the MIBBI project. Nat Biotechnol 2008; 26: 889-896 10.1038/nbt.1411 18688244 PMC2771753

[4] Campbell BJ Smith JL Hanson TE Klotz MG Stein LY Lee CK Wu D Robinson JM Khouri HM Eisen JA Adaptations to submarine hydrothermal environments exemplified by the genome of Nautilia profundicola. PLoS Genet 2009; 5: e1000362 10.1371/journal.pgen.1000362 19197347 PMC2628731

[5] Liolios K Mavromatis K Tavernarakis N Kyrpides NC The Genomes On Line Database (GOLD) in 2007: status of genomic and metagenomic projects and their associated metadata. Nucleic Acids Res 2008; 36: D475-D479 10.1093/nar/gkm884 17981842 PMC2238992

[6] Meyer F Paarmann D D'Souza M Olson R Glass EM Kubal M Paczian T Rodriguez A Stevens R Wilke A The metagenomics RAST server - a public resource for the automatic phylogenetic and functional analysis of metagenomes. BMC Bioinformatics 2008; 9: 386 10.1186/1471-2105-9-386 18803844 PMC2563014

[7] Field D Sterk P Kyrpides N Glöckner FO Hirschman L Garrity G Wooley J Gilna P Meeting Reports from the Genomic Standards Consortium (GSC) Workshops 6 and 7. *Stand. Genomics Sci*. 2009;1:68-71 10.4056/sigs.25165 PMC3035212 21304639

